# Incorporation of omega-3 polyunsaturated fatty acids into glycerolipids in microalga *Emiliania huxleyi*: radiochemical analysis of glycerolipid biosynthesis

**DOI:** 10.1128/aem.01454-25

**Published:** 2025-11-10

**Authors:** Kaiwen Sun, Dauenpen Meesapyodsuk, Xiao Qiu

**Affiliations:** 1 Department of Food & Bioproduct Sciences, University of Saskatchewan 7235, Saskatoon, Saskatchewan, Canada; 2 National Research Council of Canada 85071 , Saskatoon, Saskatchewan, Canada; Chalmers tekniska hogskola AB, Gothenburg, Sweden

**Keywords:** *Emiliania huxleyi*, microalgae, glycerolipid biosynthesis, fatty acid assembly, lipid metabolism

## Abstract

Marine microalgae are primary producers of nutritionally important ω-3 polyunsaturated fatty acids (PUFAs), such as docosahexaenoic acid (DHA, 22:6n-3), and octadecapentaenoic acid (OPA, 18:5n-3). However, how these fatty acids, after synthesis, are incorporated into glycerolipids in microalgae remains largely unknown. To investigate the assembly of fatty acids and glycerol into different glycerolipids, ^14^C-acetate and ^14^C-glycerol were used to trace their flux into glycolipids (GL) and phospholipids (PL) in the microalga *Emiliania huxleyi*. ^14^C-acetate labeling showed that freshly synthesized fatty acids were predominantly incorporated into phosphatidylethanolamine (PE) and phosphatidylcholine (PC) over monogalactosyldiacylglycerol (MGDG) and digalactosyldiacylglycerol (DGDG), with the more efficient incorporation into the sn-2 position of PL. A similar flux pattern of glycerol backbone to glycerolipids was also observed in ^14^C-glycerol labeling. Simultaneous decrease of both acyl and backbone fluxes to PE alongside the corresponding increase in PC over the labeling courses revealed the conversion of PE to PC through head group methylation. Positional analysis of labeled glycerolipids showed that saturated fatty acids (SFA) were initially found exclusively at the sn-2 position of MGDG, while OPA, not DHA, emerged in MGDG with preference at the sn-1 position. In contrast, DHA, not OPA, was incorporated into PC with preference at the sn-2 position. These results indicate that the two ω-3 fatty acids are incorporated through completely separate pathways in *E. huxleyi*. The knowledge about the biosynthesis and assembly of ω-3 PUFAs at the stereospecific positions of glycerolipids is highly valuable for developing targeted strategies in the metabolic engineering of these fatty acids in microalgae.

**IMPORTANCE:**

Marine microalgae are primary producers of nutritionally important ω-3 long-chain polyunsaturated fatty acids (PUFAs). However, how these fatty acids, after synthesis, are incorporated into glycerolipids in microalgae remains largely unknown. To investigate the assembly of fatty acids and glycerol into different glycerolipids, ^14^C-acetate and ^14^C-glycerol were used to trace their flux into glycolipids and phospholipids with two different protocols, steady-state labeling and pulse-chase labeling in *Emiliania huxleyi,* a cosmopolitan microalga that can produce a high level of docosahexaenoic acid (DHA, 22:6n-3) and octadecapentaenoic acid (OPA, 18:5n-3). Results indicate that the two ω-3 fatty acids are incorporated through completely separate pathways with high stereospecificity in *E. huxleyi*. Knowledge gained from the research is highly valuable for developing targeted strategies for the optimized production and metabolic engineering of these fatty acids in microalgae and heterologous systems.

## INTRODUCTION

Microalgae are a highly diverse group of unicellular and photosynthetic eukaryotes found in a variety of environments, including both freshwater and marine ecosystems, where they form the foundation of nutritional food chains (1, 2). In particular, they are the primary producers for nutritionally important ω-3 polyunsaturated fatty acids (PUFAs), such as docosahexaenoic acid (DHA, 22:6n-3), eicosapentaenoic acid (EPA, 20:5n-3), and octadecapentaenoic acid (OPA, 18:5n-3), essential components of membrane lipids and precursors for signaling molecules in eukaryotes (3–7).

In plants and algae, membrane lipids are mainly glycerolipids that are categorized into phospholipids (PL), including phosphatidylcholine (PC), phosphatidylethanolamine (PE), phosphatidylinositol (PI), phosphatidylglycerol (PG), and phosphatidylserine (PS), and glycolipids (GL), including monogalactosyldiacylglycerol (MGDG) and digalactosyldiacylglycerol (DGDG) (8). Generally, they are synthesized through sequential acylations of two fatty acids to a glycerol backbone at the sn-1 and sn-2 positions with a different head group at the sn-3 position. In plants, biosynthesis of glycerolipids follows two interconnected pathways: the prokaryotic pathway for biosynthesis of GLs, such as MGDG and DGDG, in plastids and the eukaryotic pathway for biosynthesis of PL, such as PC and PE, in the endoplasmic reticulum (9–13). However, the biosynthetic process of PL and GL with ω-3 PUFAs in microalgae is largely unknown.

*Emiliania huxleyi* is an abundant marine microalga in oceans that plays a crucial role in marine ecosystems and biogeochemical cycles. As a major producer of calcium carbonate coccoliths, it is substantially involved in the marine carbon cycle by influencing carbon sequestration and ocean alkalinity (14, 15). As a primary producer of ω-3 PUFAs, such as DHA and OPA, it contributes significantly to marine food chains for ω-3 nutrition. Our recent study indicates that OPA is synthesized by a plastidic aerobic pathway through sequential desaturations with the last step of Δ3 desaturation, while DHA is synthesized by an extraplastidic anaerobic pathway catalyzed by a PUFA synthase in the alga species (16). This study aims to elucidate how and where these fatty acids are assembled into different glycerolipids using steady-state and pulse-chase radioisotope labeling protocols. The results have elucidated two independent pathways for the biosynthesis and assembly of PC and MGDG with distinct fatty acid profiles in microalgae.

## RESULTS

### Tracing the acyl flux to glycerolipids by ^14^C-acetate labeling

To examine acyl flux to glycerolipids in *E. huxleyi*, ^14^C-acetate was employed to trace the incorporation of freshly synthesized fatty acids into PL and GL using two different protocols, steady-state labeling and pulse-chase labeling.

### Steady-state labeling by ^14^C-acetate

**Fig 1:**
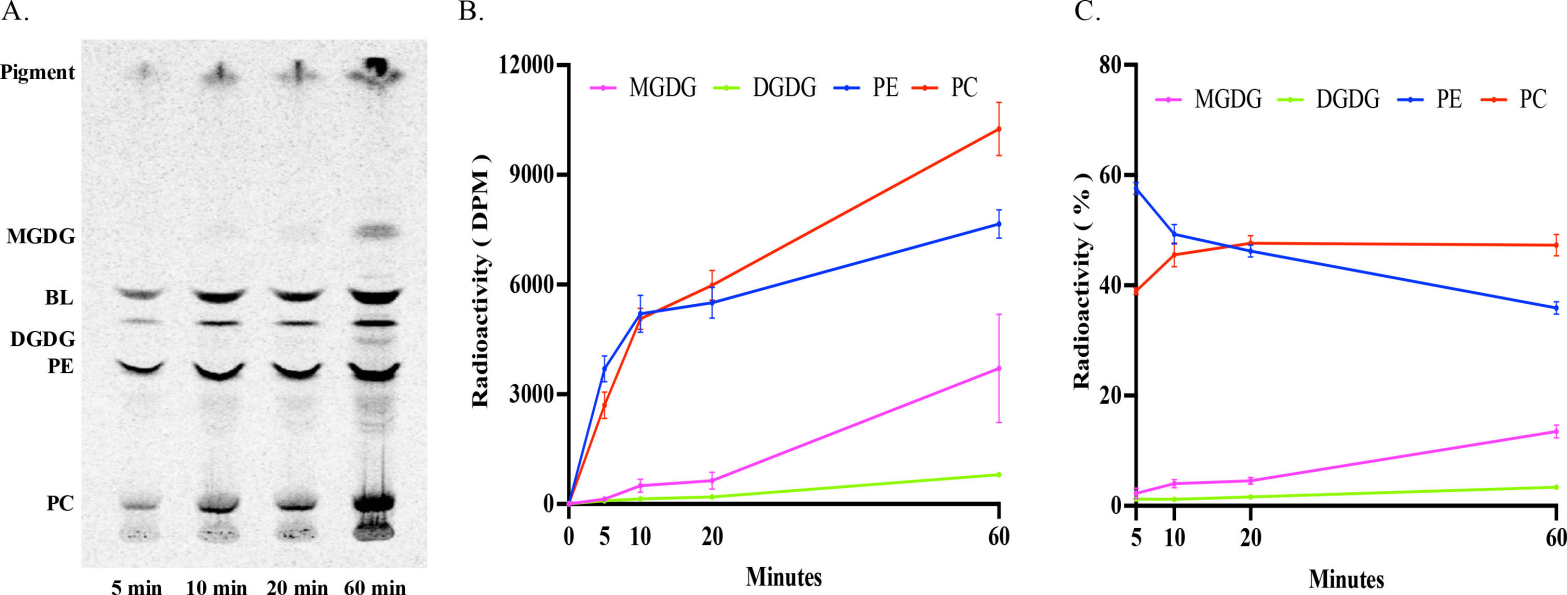
Steady-state labeling of glycerolipids in *E. huxleyi* by ^14^C-acetate in an hour time course. (**A**) Thin-layer chromatography (TLC) separation of acyl-labeled total lipids into lipid classes MGDG, BL, DGDG, PE, and PC. (**B**) Radioactivity in phospholipids/glycolipids PC, PE, MGDG, and DGDG. (**C**) Relative radioactivity of PC, PE, MGDG, and DGDG over the total labeled phospholipids/glycolipids. The values are means ± SD from three biological replicates.

To trace the initial incorporation of newly synthesized fatty acids into glycerolipids, the alga was fed with ^14^C-acetate continuously for 60 min in a steady-state labeling. The total glycerolipids at four time points in the time course (5, 10, 20, 60 min) were extracted and analyzed. As shown in Fig. 1A, labeled radioactivity was predominantly found in two phospholipids, PE and PC, followed by two betaine lipids (BL) and two glycolipids, MGDG and DGDG, over the time course. As for phospholipids/glycolipids, at 5 min of labeling, the highest level of radioactivity was detected in PE, followed by PC, while radiolabeled MGDG and DGDG were barely detectable at this time point. As labeling progressed, radioactivity in both PE and PC continued to rise, with PC increasing faster and surpassing PE after 10 min of labeling. At this time point, both MGDG and DGDG started to show measurable levels of radioactivity, with MGDG radioactivity increasing faster thereafter (Fig. 1B). By the end of the labeling course, PC was the most labeled glycerolipid, representing about 47% of the total PL and GL radioactivity, followed by PE (36%), MGDG (14%), and DGDG (3%) (Fig. 1C). These results indicate that ^14^C acetate is rapidly taken up by *Emiliania* and utilized for fatty acid synthesis, and freshly synthesized fatty acids are predominantly incorporated into PL over GL. In PLs, the decrease of acyl-labeled PE being accompanied by the increase of acyl-labeled PC over the time course (Fig. 1B) is indicative of acyl flux from PE to PC.

**Fig 2:**
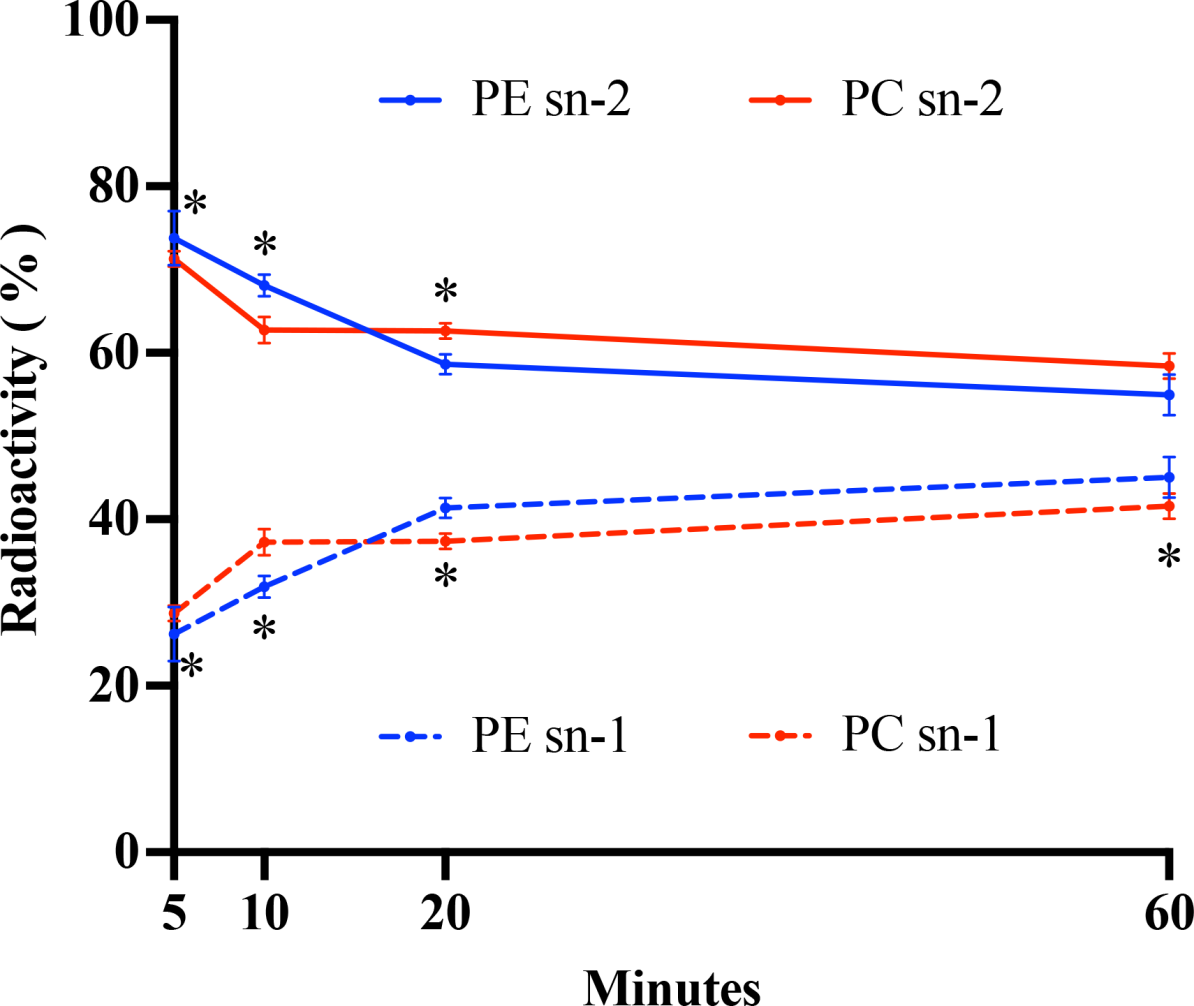
Relative radioactivity incorporated at the sn-1 and sn-2 positions of PC and PE in the steady-state labeling by ^14^C-acetate in an hour time course. The values are means ± SD from three biological replicates. Significant differences (Student’s t test, *P* value < 0.05) between sn-1 and sn-2 position of PE at each time point are indicated by asterisks above the data points; significant differences (Student’s t test, *P* value < 0.05) between sn-1 and sn-2 position of PC at each time point are indicated by asterisks below the data points.

To investigate the stereospecific incorporation of newly synthesized fatty acids into the predominantly labeled PLs, stereochemical analysis of labeled fatty acids in PC and PE across the time course was performed. As shown in Fig. 2, freshly synthesized fatty acids were preferentially incorporated into the sn-2 position of phospholipids over the time course. At 5 min of labeling, more than 70% of labeled fatty acids were found at the sn-2 position of both PE and PC. Although the proportion decreased slightly afterward, more than 55% of labeled fatty acids remained at the sn-2 position of PE and PC by the end of the labeling course. The incorporation rate of labeled fatty acids at the sn-2 position was initially higher in PE than in PC; however, as labeling progressed, the rate in PC surpassed that of PE.

### Pulse-chase labeling by ^14^C-acetate

**Fig 3:**
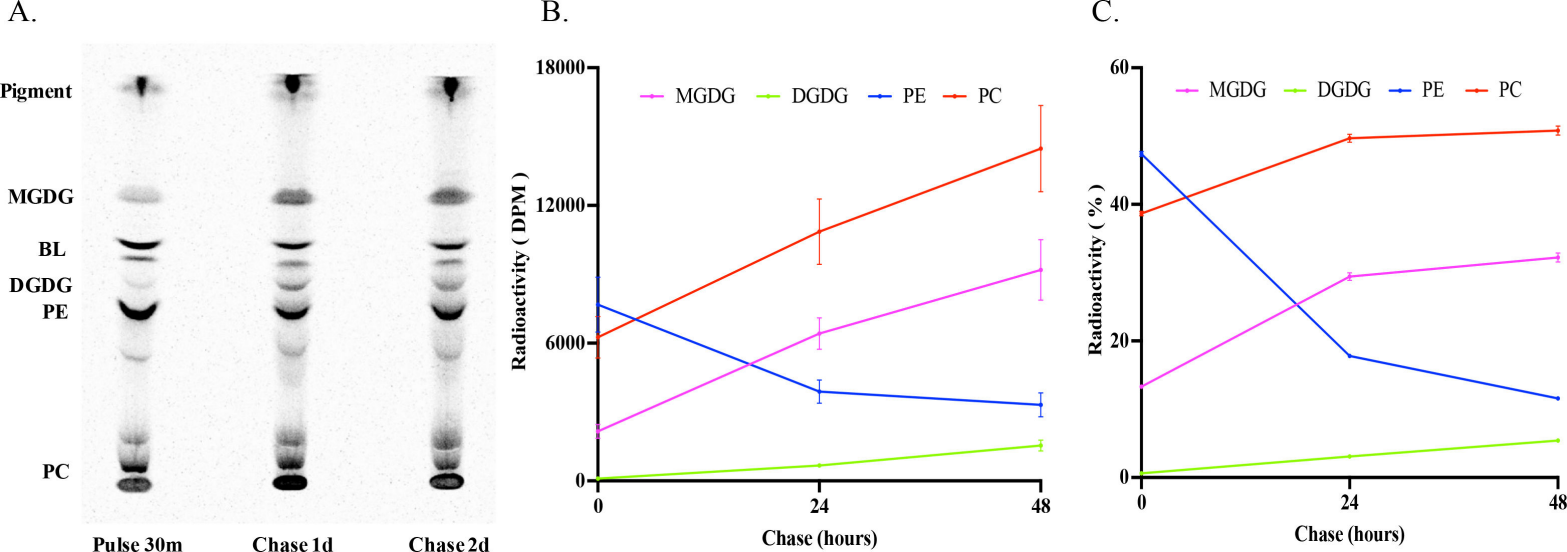
Pulse-chase labeling of glycerolipids in *E. huxleyi* by ^14^C-acetate in a pulse-chase time course. (**A**) TLC separation of acyl-labeled total lipids into lipid classes MGDG, BL, DGDG, PE, and PC. (**B**) Radioactivity in phospholipids/glycolipids PC, PE, MGDG, and DGDG. (**C**) Relative radioactivity of PC, PE, MGDG, and DGDG over the total labeled phospholipids/glycolipids. The values are means ± SD from three biological replicates.

To investigate the acyl assembling dynamics for a longer term, a pulse-chase labeling was conducted. *Emiliania* was fed with ^14^C-acetate for 30 min. Afterward, unconsumed radioisotope substrate was washed off, leaving the microalgal cells with cold acetate to grow for another 2 days. At 30 min of labeling (pulse), 24 h (chase 1), and 48 h (chase 2) post-labeling, total glycerolipids were extracted and analyzed. Like the steady-state labeling, the most abundantly labeled glycerolipids at 30 min of pulse labeling were PE and PC, followed by a BL, and MGDG and DGDG were labeled to a lesser extent (Fig. 3A), reaffirming that freshly synthesized fatty acids were primarily incorporated in PL. During the chase phase, the labeled PE declined rapidly, dropping from 47% of the incorporation rate at pulse to 12% at chase 2. In contrast, labeled PC and MGDG increased over the same course, particularly for PC, showing a rise from 39% of the incorporation rate at pulse to 51% at chase 2 (Fig. 3B and C). This rapid decrease in acyl-labeled PE alongside a corresponding increase in acyl-labeled PC in the PL pool during the chase phase confirms the occurrence of rapid acyl flux from PE to PC.

It was noted that the total radioactivity in acyl-labeled glycerolipids increased during the chase period as compared with that at pulse. This phenomenon was also observed in other similar labeling experiments (17–19). This is probably due to the delayed incorporation of the radioisotope tracer and its derivatives into lipids inside cells. At pulse, some radioisotope tracers may still stay in intermediary pools, for example, acetyl-CoA and short-chain acids. During the chase period, these intermediates can then be channeled into fatty acid synthesis and lipid assembly, thereby boosting total labeled lipid radioactivity. Additionally, ongoing fatty acid and glycerolipid biosynthesis and expansion of lipid pools during cell growth at the chase stage may also contribute to the increase of total labeled glycerolipid radioactivity.

**Fig 4:**
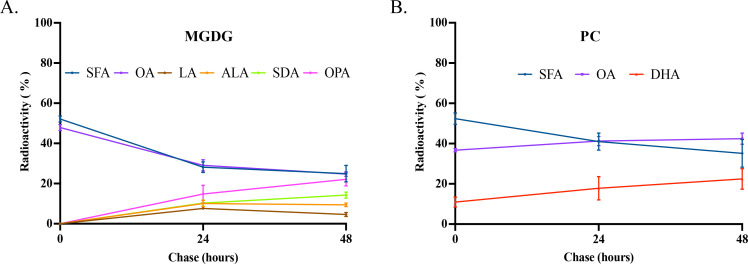
Radioactivity of labeled fatty acids in MGDG and PC in pulse-chase labeling of *E. huxleyi* by ^14^C-acetate. (**A**) Composition of radiolabeled fatty acids in MGDG. (**B**) Composition of radiolabeled fatty acids in PC. All the values are means ± SD from three biological replicates.

To obtain insights into the assembly of fatty acids into phospholipid and glycolipid, MGDG and PC, two major labeled glycerolipids with diverse fatty acid profiles, were further analyzed. As shown in Fig. 4, labeled fatty acid compositions of the two lipid classes at pulse were dominated by saturated fatty acid (SFA) and oleic acid (OA, 18:1n-9). In contrast, labeled fatty acid profiles at the two chase phases were diverse in the two lipid classes. For instance, labeled SFA and OA in MGDG at pulse accounted for 52% and 47%, respectively, with other fatty acids not detectable. During the chase periods, labeled SFA and OA declined, while four new 18C PUFAs: linoleic acid (LA, 18:2n-6), alpha-linolenic acid (ALA, 18:3n-3), stearidonic acid (SDA, 18:4n-3), and OPA (18:5n-3) were detected. Among these fatty acids, OPA was most abundant, followed by SDA, ALA, and LA (Fig. 4A). It was noted that no DHA (22:6n-3) was detected in MGDG over the entire pulse-chase course. Steady reduction of OA and corresponding increase of OPA, SDA, ALA, and LA in MGDG over the chase phase clearly indicates that OPA is synthesized in MGDG through sequential desaturations. On the other hand, SFA, OA, and DHA were three labeled fatty acids in PC at pulse. By the end of the chase period, the amount of labeled SFA decreased by 33%, while labeled OA increased by 14%, and labeled DHA increased twofold (Fig. 4B). It was noted that OPA and its immediate intermediates were completely absent in PC over the entire pulse-chase course. This result indicates that DHA, not OPA, synthesized through an anaerobic pathway (16), is exclusively incorporated into PC in the microalga.

**Fig 5:**
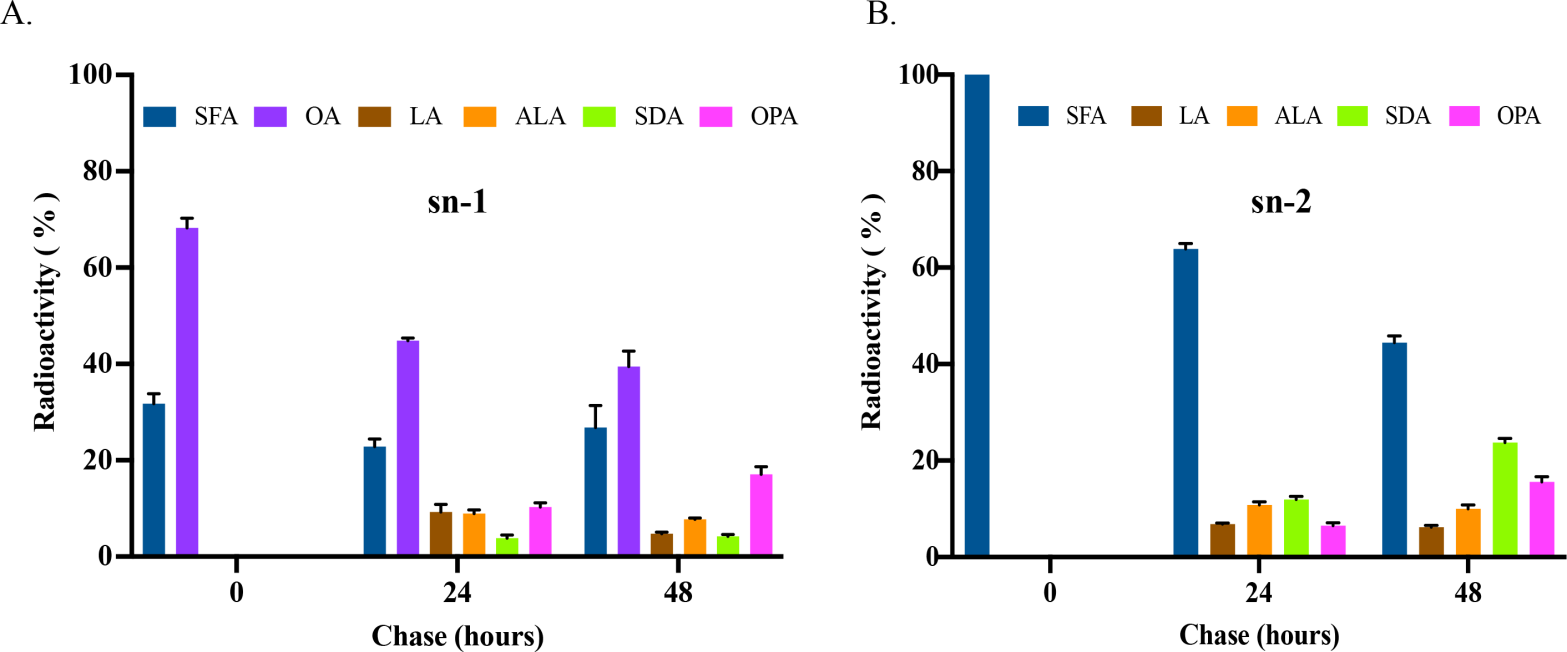
Positional analysis of labeled MGDG in pulse-chase labeling of *E. huxleyi* by ^14^C-acetate. (**A**) Composition of radiolabeled fatty acids at the sn-1 position of MGDG. (**B**) Composition of radiolabeled fatty acids at the sn-2 position of MGDG. All the values are means ± SD from three biological replicates.

To investigate the regiospecificity of the two lipid classes, the positional distribution of freshly synthesized fatty acids in MGDG and PC was determined. In MGDG at pulse, OA and SFA were predominantly located at the sn-1 position, accounting for about 68% and 32%, respectively, while SFA was exclusively present at the sn-2 position. It was noted that no 18C-PUFAs were detected at both positions of MGDG at this stage (Fig. 5A). This result indicates that MGDG is initially synthesized through the prokaryotic pathway, with SFA being exclusively allocated at the sn-2 position. In MGDG at 24 h chase, both OA and SFA declined, and LA, ALA, SDA, and OPA were all detected at the sn-1 position. At the sn-2 position, SFA declined, and LA, ALA, SDA, and OPA were also present. At the 48 h chase, OA and SFA continued to decline while OPA increased substantially at the sn-1 position. At the sn-2 position, SFA decreased further while both SDA and OPA increased continuously. It was surprisingly noted that OA was not detected at the sn-2 position of MGDG during the entire pulse-chase period (Fig. 5B). These results imply that OPA might be primarily synthesized at the sn-1 position of MGDG.

**Fig 6:**
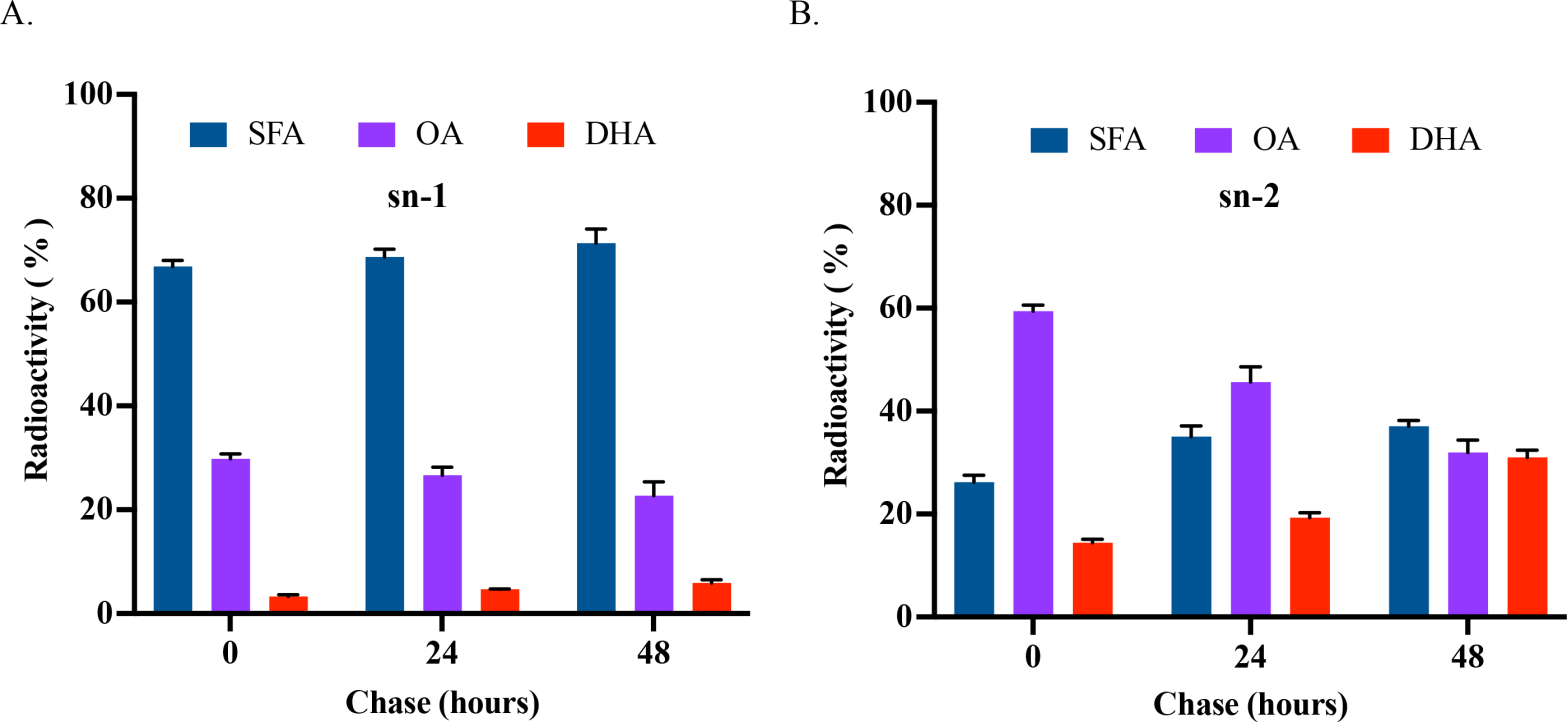
Positional analysis of labeled PC in pulse-chase labeling of *E. huxleyi* by ^14^C-acetate. (**A**) Composition of radiolabeled fatty acids at the sn-1 position of PC. (**B**) Composition of radiolabeled fatty acids at the sn-2 position of PC. All the values are means ± SD from three biological replicates.

In PC, SFA was the predominant fatty acid at the sn-1 position over the time course, accounting for about 67% at pulse and increasing slightly afterward, followed by OA, accounting for about 30% at pulse, while decreasing slightly thereafter. DHA was the least labeled fatty acid at the sn-1 position, accounting for about only 3% at pulse and increased slightly during the chase phase (Fig. 6A). At the sn-2 position, OA was the predominant fatty acid accounting for about 59% at pulse and decreased steadily over the chase phase, followed by SFA accounting for about 26% at pulse and increased slightly afterward. DHA was also the least labeled fatty acid, accounting for about 14% at pulse, but increased significantly, reaching a similar level of SFA and OA at about 31% by the end of the chase course (Fig. 6B). These results indicate that SFA was predominantly incorporated into the sn-1 position of PC while unsaturated fatty acids OA and DHA are preferentially incorporated into the sn-2 position of PC.

### Tracing the backbone flux to glycerolipids by ^14^C-glycerol

To track the flux of glycerol backbone into glycerolipids, steady-state and pulse-chase labeling of ^14^C-glycerol were also performed. During labeling, a small proportion of the supplied ¹⁴C-glycerol was converted to acetic acid for fatty acid synthesis; thus, for glycerol flux analysis, only glycerolipids with radiolabeled backbone were taken into account.

### Steady-state labeling by ^14^C-glycerol

**Fig 7:**
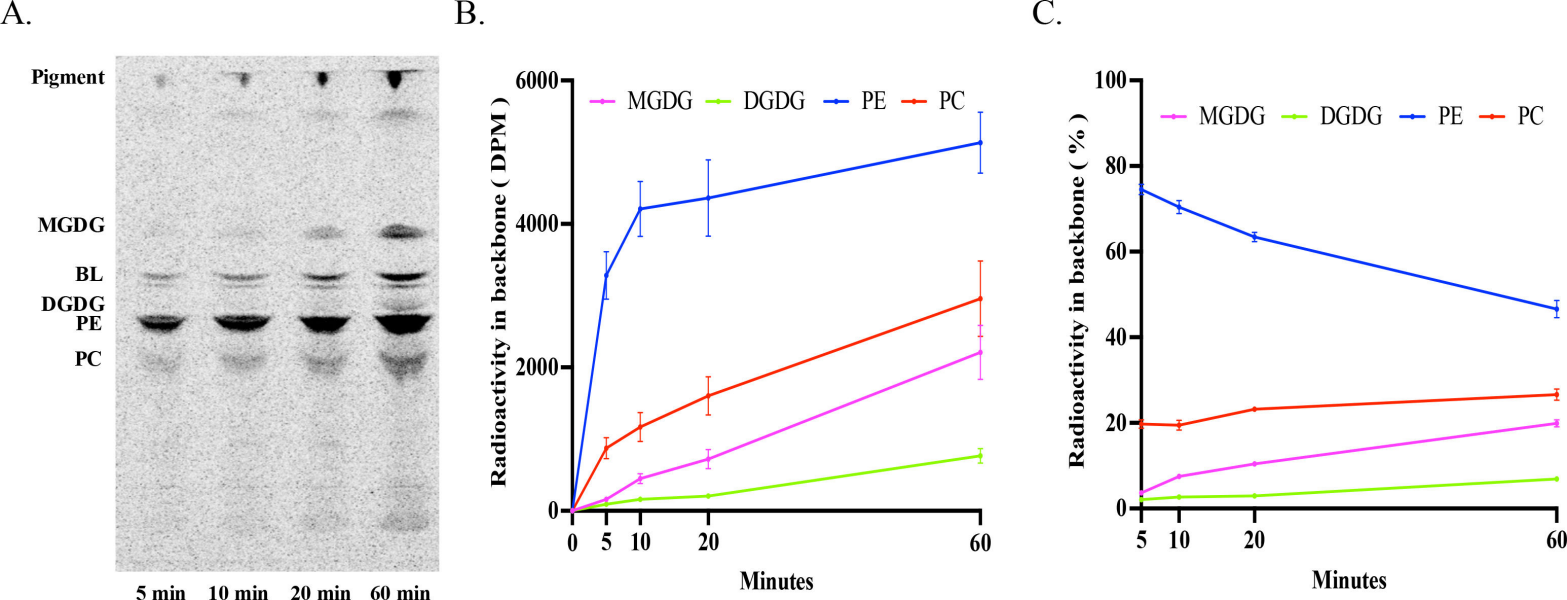
Steady-state labeling of glycerolipids in *E. huxleyi* by ^14^C-glycerol in an hour time course. (**A**) TLC separation of glycerol-labeled total lipids into lipid classes MGDG, BL, DGDG, PE, and PC. (**B**) Radioactivity in phospholipids/glycolipids PC, PE, MGDG, and DGDG. (**C**) Relative radioactivity of PC, PE, MGDG, and DGDG over the total labeled phospholipids/glycolipids. The values are means ± SD from three biological replicates.

As shown in Fig. 7A, ^14^C-glycerol was readily taken up and incorporated into the glycerol backbone of glycerolipids by *Emiliania*. At 5 min of the steady-state labeling, labeled glycerol went predominantly into PE, followed by PC and BL, with MGDG and DGDG being hardly detectable. As the labeling time progressed, radioactivity increased in phospholipids and glycolipids, but the rates of increase varied (Fig. 3B). For instance, at 5 min of labeling, PE was the predominant labeled glycerolipid, accounting for 75% of the total radioactivity of PL and GL, followed by PC (20%). Both MGDG and DGDG were labeled in significantly smaller amounts. As the labeling time progressed, the proportion of backbone-labeled PE decreased while those of backbone-labeled PC and MGDG increased, and that of labeled DGDG remained fairly stable (Fig. 7C). These results indicate that backbone flux to glycerolipids also predominantly occurs in PL. Decrease in labeled PE and concurrent increase in labeled PC over the time course imply that backbone flux also occurs from PE to PC.

### Pulse-chase labeling by ^14^C-glycerol

**Fig 8:**
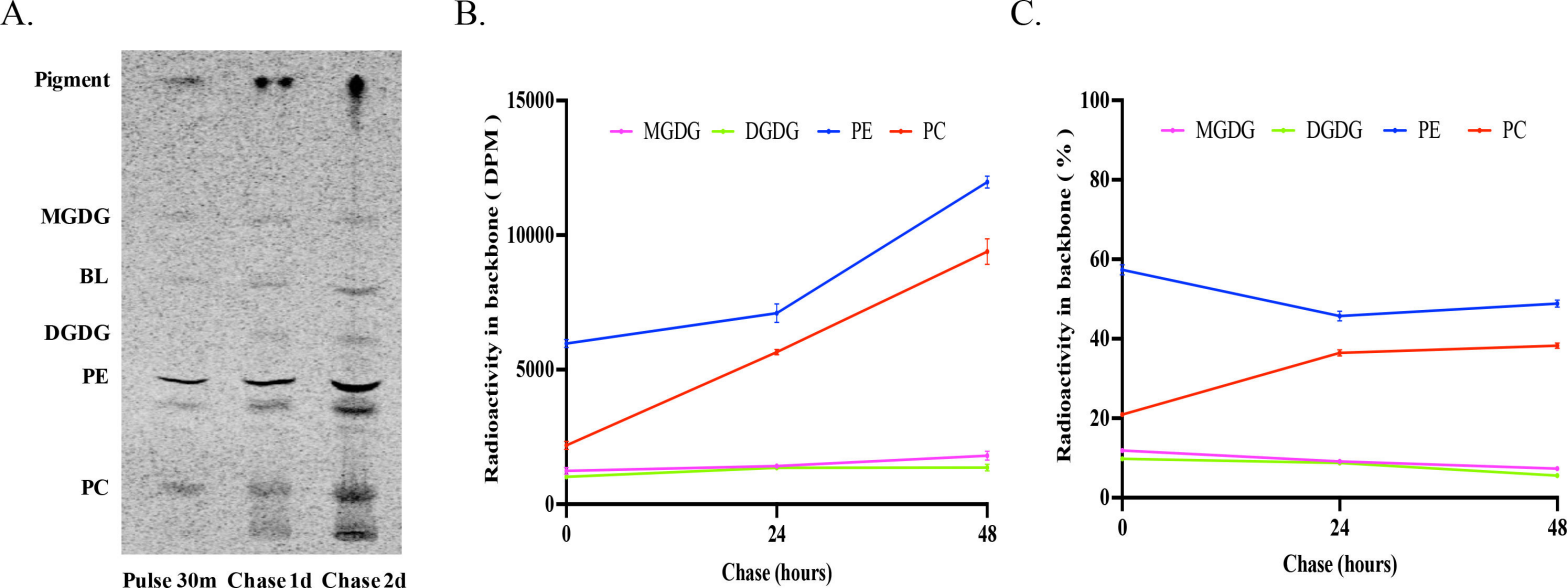
Pulse-chase labeling of glycerolipids in *E. huxleyi* by ^14^C-glycerol in a pulse-chase time course. (**A**) TLC separation of glycerol-labeled total lipids into lipid classes MGDG, BL, DGDG, PE, and PC. (**B**) Radioactivity in phospholipids/glycolipids PC, PE, MGDG, and DGDG. (**C**) Relative radioactivity of PC, PE, MGDG, and DGDG over the total labeled phospholipids/glycolipids. The values are means ± SD from three biological replicates.

As shown in Fig. 8A, radiolabeled glycerol was much more efficiently incorporated into phospholipids PE and PC than glycolipids MGDG and DGDG and BL throughout the pulse-chase course. As for phospholipids/glycolipids, radioactivity in PE and PC at pulse accounted for about 57% and 21% of the total radioactivity, respectively, with the rest in MGDG and DGDG. Over the chase period, the amount of radioactivity in both labeled PE and PC increased, but the rate of increase in PC was higher (Fig. 8B). By the end of the chase course, labeled PE accounted for about 49% while labeled PC was about 38% of the total radioactivity. On the other hand, the proportions of both labeled MGDG and DGDG remained relatively constant at about 10% over the entire pulse-chase course (Fig. 8C). The reciprocal proportional change of glycerol flux into PE and PC confirms the backbone flux from PE to PC.

## DISCUSSION

Microalgae have attracted much interest in recent years due to their potential for producing biofuel and high-value nutritional products, especially ω-3 PUFAs (20, 21). However, how these fatty acids are synthesized and assembled into glycerolipids in microalgae remains largely unknown. This study aimed to examine the incorporation of newly synthesized ω-3 PUFAs into phospholipids and glycolipids in *E. huxleyi* using radioactive tracers.

### Newly synthesized fatty acids are predominantly incorporated into phospholipids preferentially through the lysophospholipid acyltransferase-mediated pathway

When *Emiliania* was provided with a steady supply of ^14^C-acetate over an hour time course, newly synthesized fatty acids were more efficiently incorporated into PE and PC than MGDG and DGDG. At 5 min of labeling, more than 96% of the total PL and GL radioactivity was found in the two PLs, with only 3.5% in the two GLs. At 30 min of labeling, approximately 86% of newly synthesized fatty acids were incorporated into PL, and only about 14% of them went into GLs (Fig. 1 and 3). It is assumed that fed ^14^C-acetate can enter chloroplasts for the biosynthesis of SFA, such as 16:0 and 18:0, catalyzed by type II fatty acid synthase. Some of these long-chain SFA can then be desaturated into corresponding monounsaturated fatty acids by a plastidic soluble acyl-ACP Δ9 desaturase. Afterward, newly synthesized SFA and monounsaturated FA can either move out of plastids for the incorporation into PL in the cytosol or stay in plastids for the integration into GL for further modification. Both the steady-state and pulse-chase acyl-labeling showed that labeled fatty acids accumulated predominantly in PL rather than GL over the entire time courses, implying that freshly synthesized fatty acids in chloroplasts are primarily exported to the cytosol for the biosynthesis of phospholipids PE and PC in *Emiliania*.

In plants, the rapid incorporation of newly synthesized fatty acids occurs at the sn-2 position of PL through a lysophospholipid acyltransferase-mediated pathway catalyzed by lysophosphatidylcholine acyltransferase and lysophosphatidylethanolamine acyltransferase (22–25). Our stereochemical analysis of labeled PLs in *Emiliania* revealed that newly synthesized fatty acids were preferentially incorporated into the sn-2 position of both PC and PE (Fig. 2). A similar incorporation pattern was also observed in unicellular protist *Thraustochytrium* (26). In plants, acyl editing involves the constant exchange of acyl groups among PC, triacylglycerol (TAG), and the acyl-CoA pool, enabling the PUFAs produced on PC to be channeled into TAG for storage (19, 27–29). *Emiliania* produces little TAG; thus, the exact functional role of this acyl trafficking in microalgae remains to be determined.

### PC can be synthesized through methylation of PE

Tracing the acyl flux to glycerolipids by ^14^C-acetate labeling showed that the rapid decrease in acyl-labeled PE was accompanied by the corresponding increase in acyl-labeled PC in both steady-state and pulse-chase labeling (Fig. 1 and 3), indicating that acyl flux occurs from PE to PC. Tracing the backbone flux to glycerolipids by ^14^C-glycerol labeling showed a decrease in backbone-labeled PE along with a concurrent increase in backbone-labeled PC in both labeling courses (Fig. 7 and 8), indicating that the backbone flux also occurs from PE to PC. Simultaneous acyl and backbone flux from PE to PC during the labeling courses suggests that PE is converted to PC by head group modification, likely mediated by N-methylation in *Emiliana*. In eukaryotes, biosynthesis of PC mainly goes through the *de novo* process starting from the activation of head-group choline (30). However, PC can also be synthesized through the methylation of PE catalyzed by PE methyltransferase or the methylation of phosphoethanolamine to phosphocholine, a precursor for the *de novo* biosynthesis of PC, catalyzed by phosphoethanolamine-N-methyltransferase (31). In human livers, about 30% of PC is synthesized via the PE methylation, and approximately 70% of PC is synthesized via the *de novo* pathway (32). In microalga *Cyanidioschyzon merolae*, PE methylation is exclusively used for PC biosynthesis (18). In addition, the methylation of phosphoethanolamine to phosphocholine for the *de novo* synthesis of PC was also observed in microalga species (33). In *Emiliania*, both acyl and backbone steady-state and pulse-chase labeling reveal the conversion of PE to PC through the PE methylation.

### Prokaryotic and eukaryotic pathways for biosynthesis of ω-3 PUFA-containing glycerolipids operate independently

In plants, glycerolipids can be synthesized through two pathways: prokaryotic pathway in the chloroplast and eukaryotic pathway in the endoplasmic reticulum (ER). PLs, such as PC and PE, are generally synthesized through the eukaryotic pathway, while GLs, such as MGDG and DGDG, are synthesized through the prokaryotic pathway. Due to the different substrate specificity of plastidic and extraplastidic lysophosphatidic acyltransferase (LPAAT), glycerolipids synthesized through the two pathways can be distinguished by the presence of either a 16C or 18C fatty acid at the sn-2 position (34–36). Typically, GL biosynthesis in microalgae resembles that of higher plants (37, 38); however, variations have been observed across microalgal species. In *Chlamydomonas*, biosynthesis of MGDG follows the prokaryotic pathway in plants, with the presence of 16C fatty acids at the sn-2 position (39). In *Porphyridium cruentum*, very long chain PUFAs, such as EPA, initially synthesized in PC through the eukaryotic pathway, are channeled to chloroplasts for the incorporation into MGDG (40). In *Cyanidioschyzon merolae*, MGDG with 18:2 at the sn-1 position and 16:0 at the sn-2 position is synthesized through a coupled pathway (41). Because of the crosstalk between the two pathways, the occurrence of an ER-based LPAAT that prefers 16C over 18C fatty acids makes it challenging to distinguish which pathway is responsible for the synthesis of specific glycerolipids in some species (42). However, prokaryotic and eukaryotic pathways for the biosynthesis of PC and MGDG in *Emiliania* appear to occur independently from each other. Firstly, when *Emiliania* was provided with a pulse of ^14^C-acetate for 30 min, freshly synthesized 16:0 was exclusively allocated at the sn-2 position of MGDG, indicating that MGDG is initially synthesized through the prokaryotic pathway in the chloroplast. Secondly, at 24 and 48 h post-feeding, labeled 18C PUFAs increased progressively at the sn-2 position of MGDG, where no OA and DHA were detected, while these two fatty acids were predominantly found at the sn-2 position of PC (Fig. 5 and 6). Thirdly, our previous work showed that the ER-based Δ12 desaturase in *Emiliania* was inactive, rendering no LA was produced in PC when the microalga was supplied with ^14^C-labeled oleic acid (16). These imply that chloroplast-exported oleic acid is unlikely to undergo further desaturation in PC, giving 18C PUFAs that are channeled back into chloroplast for MGDG biosynthesis. All this evidence supports the notion that the prokaryotic pathway for biosynthesis of MGDG with OPA and eukaryotic pathway for biosynthesis of PC with DHA operate independently without crosstalk. However, our current results cannot absolutely rule out the possibility of diacylglycerol backbone exchange between PE and BL, and MGDG, as we lack the data on the composition and stereospecificity of fatty acids in the labeled PE and BL for confirmation due to the limited quantity of the materials over the labeling time course. In addition, it remains to be determined why OPA and DHA are incorporated through separate pathways.

When *Emiliania* was pulse-labeled with ^14^C-acetate for 30 min, SFA and OA were exclusively incorporated at the sn-1 position of MGDG. During the subsequent chase period, 18C PUFAs, including LA, ALA, SDA, and OPA, emerged along with the corresponding decline in OA and SFA at the same position. At the sn-2 position of MGDG, SFA was the only labeled fatty acid at pulse. During the chase period, the SFA level declined while the OPA level increased. Notably, OA was absent from this position of MGDG throughout the entire pulse-chase period. This result indicates that OPA is primarily synthesized through sequential desaturations at the sn-1 position of MGDG. On the other hand, DHA was only detected in PC over the entire pulse-chase course with preferential incorporation at the sn-2 position (Fig. 5 and 6).

Taken together, results from this study reveal that the biosynthesis of MGDG with OPA by the prokaryotic pathway occurs independently of the biosynthesis of PC with DHA by the eukaryotic pathway in *Emiliania*. The information on the biosynthesis and assembly of ω-3 PUFAs in the stereospecific positions of different glycerolipids is highly valuable for developing strategies in the metabolic engineering of these nutritionally important fatty acids in microalgae.

## MATERIALS AND METHODS

### Growth of *E. huxleyi*

Axenic culture of *E. huxleyi* strain CCMP1516 purchased from the National Center for Marine Algae and Microbiota (NCMA) was grown and maintained in a L1 medium without silicate (L1-Si). The algal cells were cultivated in a 500 mL Erlenmeyer flask under illumination by an LED lamp at an intensity of 100 µmol photons m^−2^ s^−1^ with a light/dark cycle of 12 h/12 h at 18°C. The growth of the alga was determined at the O.D. values at 750 nm and hemocytometer-based cell counts.

### ^14^C-acetate and ^14^C-glycerol labeling of *E. huxleyi*

Algal culture was first grown under a 12 h/12 h light/dark cycle with a light intensity of 100 µmol photons m^−2^ s^−1^ at 18°C until it reached OD_750_ at around 0.3, and then algae cells were sub-cultured by inoculating 20 mL of the culture into 180 mL of fresh medium. When the culture reached the log phase, the cells were harvested by centrifugation at 3,000 rpm at 18°C for 5 min and re-suspended in 200 mL of a fresh L1-Si medium. The cultures were then incubated at 18°C for 1 h for equilibration before adding radioisotope tracers for labeling.

For steady-state labeling of acetate, 50 μCi of [1-^14^C]-labeled sodium acetate (American Radiolabeled Chemicals, Inc., specific activity, 55 mCi/mmol) was added to a 200 mL culture. At 5, 10, 20, and 60 min of feeding, 50 mL of culture at each time point was harvested for radiochemical analysis. The same procedure was performed for steady-state labeling of glycerol, except that 100 μCi of [1,3-^14^C]-labeled glycerol (American Radiolabeled Chemicals, Inc., specific activity, 55 mCi/mmol) was added to a 200 mL culture.

For pulse-chase labeling by acetate, 50 μCi of [1-^14^C]-labeled sodium acetate (American Radiolabeled Chemicals, Inc., specific activity, 55 mCi/mmol) was added to 150 mL of *Emiliania* culture. After labeling for 30 min, 50 mL of the culture was collected for the analysis as pulse labeling, while the rest 100 mL culture was precipitated by centrifugation and washed twice with fresh medium. Afterward, the cells were resuspended in 100 mL of the L1-Si medium with the same concentration of cold sodium acetate to grow for another 2 days. Over the chase period, 50 mL of culture was collected at day 1 and day 2 post-labeling for the analysis of chase 1 and chase 2, respectively. A similar procedure was performed for pulse-chase labeling by ^14^C-glycerol. For measuring radioactivity in backbone-labeled glycerolipids from ^14^C-glycerol labeling, the lipid classes were separated into acyl-labeled and backbone-labeled fractions through transmethylation, hexane extraction, and phase separation. Radioactivity in the aqueous phase, as glycerol backbone-labeled and the hexane phase as the acyl-labeled lipids was, respectively, measured by a liquid scintillation analyzer as disintegration per minute (Tri-carb 2910 TR, Perkin Elmer, Waltham, MA, United States) (22, 43).

### Lipid extraction and fractionation

Total lipids were extracted from collected biomass following a previously reported method (26). Briefly, microalga biomass was first quenched in 80°C–85°C isopropanol with 0.01% (wt/vol) butylated hydroxytoluene, and then extracted with methanol/chloroform/water (2/1/0.8, vol/vol/vol). The organic phase containing the total lipids was collected and dried under a nitrogen gas flush, and the dry sample was resuspended in an appropriate volume of chloroform. Radioactivity in lipid extracts was determined by liquid scintillation counting (16). The total lipids were then separated into different lipid classes, including MGDG, DGDG, PC, and PE, as well as BL, on silica gel thin-layer chromatography (TLC) plates (Analtech, uniplate, 20 × 20 cm, Newark, DE, USA) using acetone/toluene/water (91/30/8, vol/vol/vol). Individual lipid classes were identified by comparison with known standards obtained from Nu-Chek-Prep, Inc. (Elysian, MN, USA) and Sigma-Aldrich (St. Louis, MO, USA). For radioactive imaging capture, TLC plates were exposed to phosphor imaging screens (Amersham Biosciences) overnight, which were then scanned by a Typhoon FLA 7000 phosphor imager (GE Healthcare Life Sciences).

### Regiochemical analysis of radiolabeled lipids

Radiolabeled lipids TLC separation was performed as described above. Individual lipid classes were scraped from the TLC plate and eluted from silica gel with chloroform/methanol/water (5/5/1, vol/vol/vol). After phase separation, the collected chloroform phase was collected and dried under a N_2_ gas flush and used for regiochemical analysis.

Regiochemical analysis of phospholipid PC was accomplished by the digestion with phospholipase A2 (PLA2) from honeybee venom (Sigma) according to previously described methods (43). PC was dissolved in 1 mL of diethyl ether and 0.1 mL of a reaction buffer containing 50 mM Tris-HCl, 5 mM CaCl_2_ (pH 8.7) with 5 units of PLA2. The reaction was mixed vigorously at room temperature for 1 h, and the ether was then evaporated under a N_2_ gas flush. The digested lipids were extracted by adding 3.8 mL of chloroform/methanol (2/1, vol/vol) and 1 mL of 0.15 M acetic acid, and the mixture was vortexed and centrifuged at 2,200 rpm for 10 min, and the chloroform phase was collected. After that, 2.5 mL of chloroform was used to back-extract the mixture, and the combined chloroform was dried under N_2_ gas. The digested lipids were separated on TLC plates developed with chloroform/methanol/acetic acid/water (50/30/8/4, vol/vol/vol/vol). Radioactivity in free fatty acids (FFA), lysophosphatidylcholine (LPC), and lysophosphatidylethanolamine (LPE) fractions was quantified by autoradiography. Regiochemical analysis of PE from ^14^C-acetate steady-state labeling was conducted essentially following the same protocol.

Regiochemical analysis of MGDG was conducted by enzymatic digestion with lipase from *Rhizomucor miehei* (Sigma), which de-acylates MGDG at the sn-1 position, producing FFAs and lysoMGDG. MGDG was first dissolved in 1 mL of diethyl ether with the lipase at a 39:1 ratio in 1 mL of a reaction buffer consisting of 50 mM boric acid and 5 mM CaCl_2_ (pH 7.8). The reaction was mixed thoroughly by vigorously shaking at room temperature for 1 h. Afterward, the ether was evaporated under N_2_, and 2 mL of methanol/chloroform (1/1, vol/vol) was added. The mixture was vortexed and centrifuged at 2,200 rpm for 10 min, and the chloroform phase containing lipids was collected. After that, the mixture was back-extracted with 2 mL of chloroform, and the combined chloroform was dried under N_2_ gas. The digested MGDG was separated on the TLC plates with acetone/toluene/water (91/30/8, vol/vol/vol). Radioactivity in FFA and lysomonogalactosyldiacylglycerol (LysoMGDG) fractions was quantified by autoradiography.

### Radiolabeled fatty acid analysis

For radiolabeled fatty acid analysis of different lipid classes, fatty acid methyl esters (FAMEs) were prepared from scraped silica gels containing individual lipid classes by heating at 80°C for 2 h with 2 mL 1% sulfuric acid in methanol (44). The sample was cooled down on ice and then added with 1 mL 0.9% NaCl and 2 mL hexane. After brief centrifugation, the hexane phase containing FAMEs was transferred to a new tube and dried under N_2_ gas flush. The dry samples were suspended in an appropriate volume of chloroform and then loaded on TLC plates pretreated with 10% AgNO_3_ in acetonitrile, which were developed with toluene/acetonitrile (97/3, vol/vol). The identity of radiolabeled fatty acids was determined by comparison with radiolabeled and cold fatty acid standards.

## Data Availability

All data sets generated for this study are included in the article.
